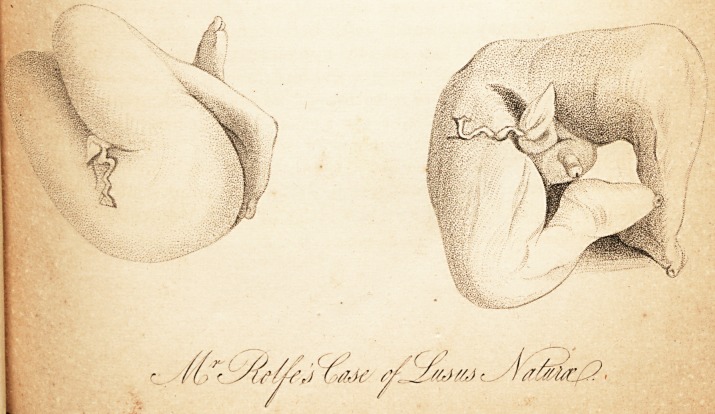# Case of Lusus Naturæ

**Published:** 1827-07

**Authors:** W. D. Rolfe

**Affiliations:** senior Surgeon-Accoucheur Extraordinary to the Bristol Dispensary, and Teacher of Midwifery at Bristol, &c. &c.


*Case of Lusus Naturæ.* Communicated by W. D. Rolfe, Esq. senior Surgeon-Accoucheur Extraordinary to the Bristol Dispensary, and Teacher of Midwifery at Bristol, &c. &c.

[WITH AN ENGRAVING.]

This lusus naturæ presents the appearance of the lower half of a male fœtus of the fifth or sixth month, truncated between the lower lumbar vertebræ. The inferior extremities are perfectly formed, excepting that there are only two toes on each foot,—viz. the great toe and the next; and that the back part of the legs are confined to the thighs for a considerable extent by the common integuments. The penis and scrotum are perfect. The remains of the umbilical cord has a truncated appearance, but on close examination is found to be covered by the integuments, excepting some very small vessels, which have been the means of communication between it and the placenta. There is no appearance of anus.

On making an incision anteriorly into the cavity of the abdomen, none of the abdominal viscera were found but the liver, which had only one lobe, and, situated superiorly, occupied by far the greater portion of the cavity. The anterior and inferior portion was filled by the bladder, which extended upwards to the umbilical cord; its longitudinal diameter very much exceeded that of its transverse.

The blood-vessels were unusually small.

Before an incision was made, the integuments covered the whole of the fœtus uninterruptedly, excepting at the point of the penis and at the opening of the urethra. In the hollow of the sacrum there was found a trifling development of the spinal marrow.

*26th May*, 1827.

## Figures and Tables

**Figure f1:**